# Strategies for spinal surgery reimbursement: bundling in the working-age population

**DOI:** 10.1186/s12913-021-06112-0

**Published:** 2021-02-02

**Authors:** Michael K. Dalton, Christer Mjåset, Adoma Manful, Melvin D. Helgeson, William Wynn-Jones, Zara Cooper, Tracey P. Koehlmoos, Joel S. Weissman

**Affiliations:** 1 Center for Surgery and Public Health, Harvard Medical School, Harvard T. H. Chan School of Public Health, Department of Surgery, Brigham and Women’s Hospital, 1620 Tremont Street, 1 Brigham Circle, Boston, MA 02120 USA; 2 Department of Health Policy and Management, Harvard T. H. Chan School of Public Health, 677 Huntington Ave, Boston, MA 02115 USA; 3 Research and Communication Unit for Musculoskeletal Health (FORMI), Oslo University Hospital, PO Box 4950, Nydalen, 0424 Oslo, Norway; 4 Commonwealth Fund Harkness Fellowship, 1 East 75th Street, New York, NY 10021 USA; 5 Department of Orthopaedics, Walter Reed National Military Medical Center, 8901 Wisconsin Ave, Bethesda, MD 20814 USA; 6 F. Edward Hébert School of Medicine, Uniformed Services University of the Health Sciences, 4301 Jones Bridge Road, Bethesda, MD 20184 USA

**Keywords:** Spine surgery, Bundled payments, Episode of care, Payment reform

## Abstract

**Introduction:**

Bundled payments for spine surgery, which is known for having high overall cost with wide variation, have been previously studied in older adults. However, there has been limited work examining bundled payments in working-age patients. We sought to identify the variation in the cost of spine surgery among working age adults in a large, national insurance claims database.

**Methods:**

We queried the TRICARE claims database for all patients, aged 18–64, undergoing cervical and non-cervical spinal fusion surgery between 2012 and 2014. We calculated the case mix adjusted, price standardized payments for all aspects of care during the 60-, 90-, and 180-day periods post operation. Variation was assessed by stratifying Hospital Referral Regions into quintiles.

**Results:**

After adjusting for case mix, there was significant variation in the cost of both cervical ($10,538.23, 60% of first quintile) and non-cervical ($20,155.59, 74%). Relative variation in total cost decreased from 60- to 180-days (63 to 55% and 76 to 69%). Index hospitalization was the primary driver of costs and variation for both cervical (1st-to-5th quintile range: $11,033–$19,960) and non-cervical ($18,565–$36,844) followed by readmissions for cervical ($0–$11,521) and non-cervical ($0–$13,932). Even at the highest quintile, post-acute care remained the lowest contribution to overall cost ($2070 & $2984).

**Conclusions:**

There is wide variation in the cost of spine surgery across the United States for working age adults, driven largely by index procedure and readmissions costs. Our findings suggest that implementing episodes longer than the current 90-day standard would do little to better control cost variation.

**Supplementary Information:**

The online version contains supplementary material available at 10.1186/s12913-021-06112-0.

## Introduction

The United States (US) has long had the most expensive healthcare system per capita and healthcare expenditures continue to rise, without a demonstrable corresponding improvement in health outcomes [1, 2]. Among the recognized drivers of the ballooning healthcare costs are fragmentation of care, variation in costs at regional and provider level and variation in clinical practice [1]. In order to control the ever increasing amount spent on healthcare, several value based payment models have been proposed, one such model was bundled payments [3, 4].

Bundled payment models, such as the Centers for Medicare and Medicaid Services (CMS) Bundled Payments for Care Improvement (BCPI), offer a standardized payment for all healthcare provided during a defined time period, termed an “episode of care” [5]. Surgical care has been a prime target for bundled payments, due to variable costs though all phases of care and an easy to identify start date [6, 7]. Several studies have shown that surgical bundles in Medicare have led to real or potential cost savings with no decrease in quality [8–13], including bundles for spine surgery [14]. Recent work by Wynn-Jones, et al., examined the potential impact of a bundled payment model on the cost of common surgical procedures among a working age adult population (age 18–64) including spinal surgery [15]. They found significant variation in overall costs and that spending patterns in the different phases of care for working age adults (including index hospitalization, readmissions, post-acute care, and physician fees) diverged from those observed in the older adult Medicare population.

Spine disorders are associated with high healthcare spending across the adult age spectrum in the US, making them a particularly attractive target for value based payment strategies, with one study finding that those with spine conditions had healthcare expenditures 73% higher than those without spine disorders [16]. Other work utilizing national secondary data has shown that more than 400,000 primary spinal fusion operations are performed annually in the US and that the number of spine operations performed annually has been consistently increasing, with approximately half of patients requiring these operations under age 65 [17–19].

Prior studies of bundled payment models in spine surgery have largely examined the 65-and-over Medicare population and focused on fixed 90-day episodes of care [14]. In our study, we evaluated variation in costs for spinal fusion surgery among working age adults to show the potential impact of implementing bundled payments in this population and identify drivers of variation in the cost of spine surgery. We also sought to identify variation in cost by indication for the procedure and to establish the optimal episode of care time period for spine surgery.

## Methods

### Study data and population

We employed data from the TRICARE claims in the Military Health System Data Repository. TRICARE is the US Department of Defense health insurance product that covers members of the armed forces, retirees, and their dependents; approximately 80% are not active duty servicemembers [20]. The population covered by TRICARE has been shown to be nationally and demographically representative of US population under 65 years of age [20, 21]. The US Military Health System (MHS) is a bifurcated care system, providing care at both military owned hospitals (direct care) and at civilian hospitals where TRICARE functions as a fee-for-service health insurance product (purchased care). Importantly, this system does not include care provided by the US Department of Veterans Affairs. This study examined data from TRICARE Prime, one of TRICARE’s constituent insurance plans.

Patients who underwent spinal fusion operations between 2012 and 2014 were identified by International Classification of Diseases ICD, Ninth Revision, Clinical Modification (ICD-9-CM) Procedure Codes and Medicare Severity-Diagnosis Related Group (MS-DRG) Codes. Patients were stratified by procedure category: cervical spinal fusion and non-cervical spinal fusion (Additional file 1). Procedures performed for trauma, fracture, or malignancy were excluded. Demographic information, including age, sex, race, and the sponsor beneficiary’s military rank, was collected. Sponsor rank has been previously used as a proxy for socio-economic status, with junior enlisted sponsor rank being considered indicative of lower socioeconomic status [22, 23]. For patients with missing data regarding race, their sponsor beneficiary’s race was used.

In order to maximize our ability to capture all costs, patients with dual coverage with another payer were excluded. Patients who were admitted directly from skilled nursing, rehabilitation, or hospice facilities or who died during the index hospitalization were excluded in order to create a more homogenous cohort where we could focus on regional cost variation. As in prior studies, we used Hospital Referral Regions (HRR) as our aggregated unit of analysis [15, 24]. HRRs where fewer than 5 cases were performed were excluded from this analysis.

### Calculation of Tricare payments

We used payments made by TRICARE on behalf of each patient to commercial healthcare providers rather than submitted charges by the hospitals. We collected information on all payments made on behalf of each patient during the bundle period, including those for inpatient and outpatient services. We calculated payments made for all claims during episodes of care of varying duration (60-, 90-, and 180-day). All patient specific payment information for the 6 months prior to the index admission was used in calculation of the case mix index.

We applied a regional price standardization, similar to those used in Medicare, to control for claims cost variation resulting from variation in wage indices [25, 26]. Factors included in case mix adjustment were patients’ age, sex, race, sponsor rank, and admission acuity. We also included each patient’s Charlson Comorbidity Index to account for pre-existing comorbid conditions and TRICARE claims payments made in the 6 months prior to the index procedure.

Each payment was categorized into one of four payment buckets. The Index Hospitalization includes claims paid by TRICARE for the reference inpatient procedure. Readmission payments include claims paid for inpatient acute-care hospitalizations and procedures performed after discharge from the index hospitalization but within the bundle period. Post-acute care payments included those for acute or sub-acute rehabilitation facilities, skilled nursing facilities, outpatient services, home healthcare, hospice, durable medical equipment, as well as facility fees for outpatient care. Healthcare Professional Fees constituted the fourth category.

### Statistical methods

Case mix adjustment was done using a generalized linear mixed model using a log link function. We included a random effect for the grouping variable HRR, to account for correlation of outcomes within an HRR, and assumed a gamma distribution based on prior literature on modelling cost data. From this, we obtained a case-mix and empirical Bayes reliability-adjusted estimate of the mean cost for each HRR. HRRs were sorted by the adjusted total payments made for the period from discharge from index hospitalization to the end of the episode of care duration. The payments were grouped into quintiles and the average per quintile reported with confidence intervals. Consistent with previous work, inter-quintile variation was measured by using the inter-quintile difference, defined as the absolute difference in dollars between the lowest and highest quintile, expressed as a percentage of the lowest quintile [15]. The HRRs were then resorted, grouped into quintiles and averaged, for each of the four payment buckets identified (i.e., Index Hospitalization payments, Readmission payments, Post-acute Care payments, and Healthcare Professional fees). This process was repeated each spinal fusion type and episode of care duration combination. (i.e., cervical vs noncervical at 60-, 90-, and 180-days).

Finally, we also followed each patient for 1 year after their index procedure to identify patients undergoing reoperation beyond what was captured by the 180-day bundle episode. This was done in order to test whether our longest episode duration (180-days) was sufficient to capture major sources of cost variation related to the index procedure in this population, or if delayed reoperations would mandate even longer episode durations to properly account for cost variability.

A *p*-value less than 0.05 was a priori determined to be statistically significant. All analyses were performed using SAS version 9.4. The study data were used under a data use agreement with the Uniformed Services University of the Health Sciences, and the study was deemed exempt from full review by the institutional review board of Uniformed Services University of the Health Sciences and the Partners Healthcare Human Research Committee.

## Results

We identified 12,811 patients meeting inclusion criteria, of which 6977 underwent a cervical spine fusion and 5834 underwent a non-cervical spinal fusion. These procedures were performed for TRICARE beneficiaries in 168 HRRs during the study period and were included in this analysis.

Total costs for non-cervical spinal operations were higher than total costs for cervical spine operations (Table 1). There was significant variation in the price standardized and case mix adjusted (fully adjusted) amount paid by TRICARE for both cervical and non-cervical spinal fusions. The inter-quintile variation in the fully adjusted price for non-cervical spinal fusions was higher than for cervical spinal fusions across 60- (76% vs 63%), 90- (74% vs. 60%), and 180-day (69% vs 55%) episodes, although the difference narrowed with increasing episode duration.

**Table 1:** Actual, price-standardized, and fully adjusted TRICARE payments during 60-, 90- and 180-day episode lengths for cervical and non-cervical spinal fusion

DRG	Length of Bundle	Method of Cost Calculation	Quintile					Difference* (% of 1st Quintile)
			1	2	3	4	5	
Cervical	60 day bundle	*Actual*	$16,012	$17,760	$19,118	$21,512	$29,136	$13,124 (82%)
		*Price-Standardized*	$16,671	$18,084	$19,203	$21,341	$27,562	$10,891 (65%)
		*Price Standardized + Casemix Adjusted*	$16,728	$18,059	$19,112	$21,236	$27,281	$10,553 (63%)
	90 day bundle	*Actual*	$16,677	$18,472	$19,929	$22,420	$30,259	$13,581 (81%)
		*Price-Standardized*	$17,389	$18,865	$20,061	$22,166	$28,532	$11,143 (64%)
		*Price Standardized + Casemix Adjusted*	$17,459	$18,804	$19,960	$22,077	$27,997	$10,538 (60%)
	180 day bundle	*Actual*	$18,575	$20,560	$22,426	$24,884	$33,528	$14,953 (81%)
		*Price-Standardized*	$19,483	$20,949	$22,616	$24,550	$31,129	$11,646 (60%)
		*Price Standardized + Casemix Adjusted*	$19,570	$20,880	$22,309	$24,377	$30,261	$10,691 (55%)
Non-cervical	60 day bundle	*Actual*	$25,879	$29,324	$32,028	$35,551	$49,211	$23,331 (90%)
		*Price-Standardized*	$27,130	$29,915	$32,337	$35,341	$46,942	$19,812 (73%)
		*Price Standardized + Casemix Adjusted*	$26,617	$29,722	$32,083	$34,927	$46,784	$20,168 (76%)
	90 day bundle	*Actual*	$26,566	$30,128	$32,952	$36,573	$50,734	$24,167 (91%)
		*Price-Standardized*	$27,955	$30,750	$33,237	$36,139	$47,640	$19,685 (70%)
		*Price Standardized + Casemix Adjusted*	$27,410	$30,661	$33,063	$35,894	$47,565	$20,156 (74%)
	180 day bundle	*Actual*	$28,737	$32,614	$35,553	$39,436	$54,443	$25,705 (89%)
		*Price-Standardized*	$30,216	$33,420	$35,870	$38,995	$50,706	$20,490 (68%)
		*Price Standardized + Casemix Adjusted*	$29,625	$33,143	$35,622	$38,317	$50,045	$20,420 (69%)

*Difference reflects the variation between highest and lowest quintiles, shown as a dollar value and as a percentage of the lowest quintile value

The index hospitalization contributed the most to the overall cost of both cervical and non-cervical spinal operations (Table 2). At 90 days, readmissions did not contribute to the overall cost in the lowest quintiles for both types of operation and therefore had large variation. Post-acute care costs had the smallest contribution to the overall costs for both types of operation (3% in quintile 1 to 10% in quintile 5).

**Table 2:** TRICARE price standardized and case mix adjusted payments by payment bucket for simulated 90-day episodes of care after cervical and non-cervical spinal fusion

Simulated Bundle	Payment Bucket	Quintile					Difference*
		1	2	3	4	5	
Cervical 90-day Episode of Care	*Index Hospitalization*	$11,033	$11,673	$12,411	$13,670	$19,960	$8927
	*Readmissions*	$0	$0	$8912	$9786	$11,522	$11,522
	*Hospital Professional Fees*	$5027	$5752	$6274	$6898	$8056	$3029
	*Post-acute Care*	$399	$571	$702	$959	$2070	$1672
Non-Cervical 90-day Episode of Care	*Index Hospitalization*	$18,565	$21,372	$22,772	$25,749	$36,844	$18,278
	*Readmissions*	$0	$7373	$8618	$10,120	$13,933	$13,933
	*Physician Fees*	$6435	$7327	$7797	$8291	$9538	$3103
	*Post-acute Care*	$557	$851	$1153	$1540	$2985	$2427

*Difference reflects the variation between highest and lowest quintiles

A total of 787 (6.1%) patients underwent reoperation within 1 year of their index operation, of whom 357 (45.4%) had their first reoperation within 180-days of their index operation. There were 386 cervical patients that underwent reoperation and 401 non-cervical. The mean time to first reoperation was 187 days (SD 109 days). A very small number (39; 0.30%) of patients underwent multiple reoperations within 1 year.

## Discussion

In this study, we examined costs of variable length episodes for working patients undergoing spine surgery for in a working-age adult population across the United States and found significant variation in the cost of both cervical and non-cervical spine surgery. However, even though overall costs and the absolute inter-quintile difference increased as the episode duration increased, the relative inter-quintile variation decreased. We also demonstrated that for this younger population, the post-acute care spending component is only a small portion of the total spent during an episode of care, leading to relatively flat accumulation of costs in varying episode durations compared to the older adult populations.

As episode-based payment systems have evolved for patients over 65 insured by Medicare, interest has grown about the application to younger patients. Large companies, who are significant purchasers of healthcare in the US, are beginning to negotiate prospective bundled payments for various operations (including spine surgery) with specific health-systems and providers [27]. Early data from these programs have shown reduced cost, streamlined care, and presented opportunities for “hardwired” quality improvement metrics. Some commercial insurers are also beginning to design and implement bundled payment model for their beneficiaries [28]. While episode of care based bundled payments have been implemented in some specific markets, our study shows the potential impact on cost variation for spine surgery if bundled payments were implemented on a national level.

Our findings of high variation and low post-acute care spending are consistent with prior studies of episode of care-based bundles in working age adults. A recent study examining spine surgery in working age adults with commercial insurance also found significant cost variation within and between MS-DRGs, but little difference in spending over the duration of 30-, 60-, and 90-day simulated bundles [29]. The variation in cost within MS-DRGs may result from considerable differences previously demonstrated in both the underlying pathology and procedural complexity within MS-DRGs [30]. Non-cervical spinal fusions represent a more heterogenous mix of surgical approaches and complexity than do those of the cervical spine, which likely contributes to the higher variation seen in index admission costs. However, our findings vary considerably from studies of bundle payments in Medicare, in which post-acute care spending is a considerable driver of both total costs and variation in costs [31, 32]. This is consistent with prior research showing the likelihood of discharge to an inpatient rehabilitation center or skilled nursing facility significantly increases for older patients [33].

Our results show that there is wide variation in the readmission costs among patients and only a small number of patients underwent reoperation within 6 months of their index procedure. Prior studies have shown low overall rates of 30-day readmissions (5%) in spine surgery patients, with the majority being related to the index initial surgery, such as wound complications [34, 35]. Working age adults are generally healthier than the older adult Medicare population, which may account for relatively flat spending over time, as they are less likely to accrue medical costs due to unrelated conditions. While rates of reoperation and readmission are already low in this population, implementation of bundles has the ability to change behavior and care practices in ways that are not accounted for by our simulated bundles. When United Healthcare implemented its “Spine and Joint Solution,” a bundled payment for knee, hip, and spine surgeries, they found a 3.4% decrease in complications and a 10% decrease in readmissions for spine surgeries [28]. Similarly, other studies have shown that after implementation of bundled payments by Medicare, discharge to post-acute care also decreased [36, 37].

### Limitations

First, as this study utilizes claims data, it may not fully capture the complete premorbid state of the patient, potentially affecting our adjustments for case mix. Similarly, patients may pay for some services out-of-pocket or in ways not captured by claims data, though it is unlikely that these expenses contribute greatly to overall costs or variation as patients in our study have a consistent insurance product. It is also unlikely for patients to pay for elements that drive costs (such as inpatient hospitalizations and long-term care) out of pocket. We also excluded patients with evidence of other insurance coverage, such as Medicare. Due to the constraints of our data use agreement, we report our results at the HRR level rather than the hospital/institution level, though facility level work presents an opportunity for future work. While MS-DRGs are a standard grouping system for conditions and procedures and are currently used for reimbursement purposes, there is varying heterogeneity within groups that limits comparisons between MS-DRGs. In this study, we create a case mix adjustment in an attempt to control for pre-operative patient characteristics, however the optimal way of risk adjusting that best considers the severity of condition, procedural difficulty, as well as comorbidities for spine surgery remains has not yet been established [38]. Additionally, our study utilizes ICD-9-CM coding, which may limit the direct applicability of our findings given the change to ICD-10-CM coding in the US, if care patterns have changed.

## Conclusions and implications

The large variation in the cost of care for spinal surgery in working age adults indicates that there is a clear opportunity to implement a value-based payment model for these types of procedures. Unlike in studies of older adults, post-discharge spending following spine surgery among working age adults is low and does not increase substantially over the 6-months post discharge period. This suggests that increasing the episode length beyond to current standard of 90-days would do little to better control the variation in costs related to spine surgery among working age adults.

## Supplementary Information

**Additional file 1.** Episode Selection.

## Data Availability

The data that support the findings of this study are available from the United States Defense Health Agency. Restrictions apply to the availability of these data, which were used under federal Data User Agreements for the current study, and so are not publicly available.

## Abbreviations

HRR: Hospital Referral Regions
ICD-9-CM: International Classification of Diseases ICD, Ninth Revision, Clinical Modification
MS-DRG: Medicare Severity - Diagnosis Related Group
US: United States
