# Supplementary material for: Strategies for spinal surgery reimbursement: bundling in the working-age population
Source: BMC Health Serv Res. 2021 Feb 2;21:112. doi: 10.1186/s12913-021-06112-0 (PMC7852105; doi:10.1186/s12913-021-06112-0)
Supplement: Supplementary file 1 — Additional file 1. Episode Selection. [file 12913_2021_6112_MOESM1_ESM.docx]

**Additional File 1:** Episode Selection

A.1) ICD-9-CM Procedure Codes Used to Identify Index Procedures:

| ICD-9-CM  Procedure Code | Description |
| --- | --- |
| 03.09 | Other Exploration and Decompression of Spinal Canal |
| 81.0* | Spinal Fusion |
| 81.3* | Refusion Of Spine |
| 84.51 | Insertion Of Interbody Spinal Fusion Device |
| 84.59 | Insert Of Other Spinal Devices |
| 84.6* | Replacement Of Spinal Disc |
| 84.8* | Insertion, Replacement And Revision Of Posterior Spinal Motion Preservation Device(s) |

* indicates code expanded to all following digits

A.2) Medicare Severity-Diagnosis Related Group (MS-DRG)

| MS-DRG | Description |
| --- | --- |
| 471-473 | Cervical spinal fusion |
| 459-460 | Spinal Fusion (non-cervical) |
